# Vasoplegic syndrome in cardiac surgery: role of synergism between polymorphism of tumor necrosis factor beta and plasminogen activator inhibitor type 1

**DOI:** 10.1186/cc14279

**Published:** 2015-03-16

**Authors:** JL Iribarren, J Jiménez, N Perez, M Brouard, R Perez, E Hurtado, S Diosdado, M Buitrago, A Arbesu, R Martinez, M Mora

**Affiliations:** 1 Hospital Universitario de Canarias, La Laguna, Spain

## Introduction

Cardiopulmonary bypass can lead to postoperative hemodynamic disorders. Several genetic polymorphisms have been studied in this setting. We investigated the possible existence of a synergism between polymorphisms of plasminogen activator inhibitor type 1 (PAI-1) and tumoral necrosis factor beta (TNF-B) on hemodynamic response after cardiac surgery

## Methods

We prospectively studied the association between hemodynamic response and polymorphisms of TNF-B and PAI-1 in 563 patients undergoing elective cardiac surgery during the years 2008 to 2011. We tested the Hardy-Weinberg equilibrium in the sample. V18 SPSS was used.

## Results

**Figure 1:**
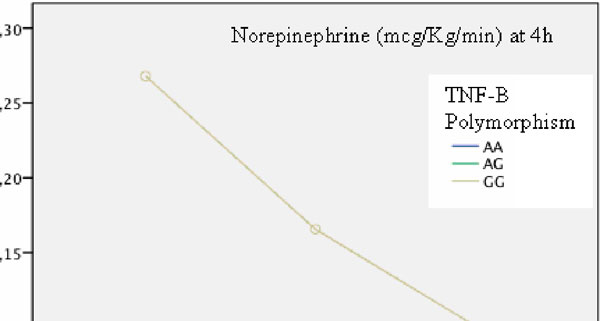


We studied 563 patients. We found significant differences in TNF-B polymorphisms regarding norepinephrine requirements at 4 hours (F: 15.9; *P *< 0.001), *post hoc *Scheffé (GG vs. AA, 0.32 (0.11 to 0.65) vs. 0.06 (0.04 to 0.09) μg/kg/minute, *P *< 0.001; GG vs. AG, 0.32 (0.11 to 0.65) vs. 0.06 (0.03 to 0.08), *P *< 0.001)) and at 24 hours (F: 8; *P *= 0.005), *post hoc *Scheffé (GG vs. AA, 0.27 (0.01 to 0.52) vs. 0.10 (0.06 to 0.14), *P *= 0.019; GG vs. AG, 0.27 (0.01 to 0.52) vs. 0.07 (0.04 to 0.09), *P *= 0.003)). Unfavorable TNF-B (G homozygous vs. allele A) and PAI-1 unfavorable (4G homozygous vs. allele 5G) were grouped, after adjusting for perioperative significant variables. The homozygous GG and 4G alleles were significant for NA 4 hours (F: 5.5; *P *= 0.02 and F: 4.1; *P *= 0.04, respectively) and GG-4G allele interaction (F: 6; *P *= 0.01) (Figure 1), while for NA at 24 hours statistics showed GG (F: 3.2; *P *= 0.07), 4G allele (F: 2; *P *= 0.15) and interaction (F: 3.6, *P *= 0.05).

## Conclusion

GG homozygous polymorphism TNF-B is associated with an increased dependence on norepinephrine after cardiopulmonary bypass, showing a synergistic action with the 4G allele of PAI-1.

